# Prognostic value of preoperative lymphocyte-related systemic inflammatory biomarkers in upper tract urothelial carcinoma patients treated with radical nephroureterectomy: a systematic review and meta-analysis

**DOI:** 10.1186/s12957-020-02048-7

**Published:** 2020-10-23

**Authors:** Yuan Shao, Wenxia Li, Dongwen Wang, Bo Wu

**Affiliations:** 1 Department of Urology, First Hospital of Shanxi Medical University, Taiyuan, 030001 Shanxi People’s Republic of China; 2 Shanxi Medical University, Taiyuan, 030001 Shanxi People’s Republic of China; 3 National Cancer Center/National Clinical Research Center for Cancer/Cancer Hospital & Shenzhen Hospital, Chinese Academy of Medical Sciences and Peking Union Medical College, Shenzhen, 518116 People’s Republic of China

**Keywords:** Upper tract urothelial carcinoma, Neutrophil-to-lymphocyte ratio, Platelet-to-lymphocyte ratio, Monocyte-to-lymphocyte ratio, Biomarker, Prognosis, Meta-analysis

## Abstract

**Background:**

Growing evidence shows that the preoperative lymphocyte-related systemic inflammatory biomarkers are associated with the prognosis of patients with upper tract urothelial carcinoma (UTUC). These markers include neutrophil-to-lymphocyte ratio (NLR), platelet-to-lymphocyte ratio (PLR), and monocyte-to-lymphocyte ratio (MLR). However, these findings are inconsistent, and the prognostic significance of these biomarkers is unclear. Moreover, the currently available prognostic indicators do not precisely predict the outcome of UTUC patients. This motivated us to investigate the prognostic values of NLR, PLR, and MLR in UTUC patients treated with radical nephroureterectomy (RNU).

**Methods:**

We prospectively registered this in PROSPERO (CRD42020186531). We performed a comprehensive literature search of the PubMed, Web of Science, EMBASE, and Cochrane Library databases to identify the eligible studies evaluating the prognostic values of preoperative NLR, PLR, and MLR. Hazard ratios with 95% confidence intervals of overall survival (OS), cancer-specific survival (CSS), disease-free survival (DFS), recurrence-free survival (RFS), metastasis-free survival (MFS), and progression-free survival (PFS) were extracted from the multivariate analyses and analyzed with fixed or random effects models when applicable. Heterogeneity among the studies was evaluated using Cochran’s Q test and *I*^2^ statistic. Sensitivity and subgroup analyses were conducted to explore the origin of heterogeneity. The Newcastle-Ottawa Scale (NOS) was applied to assess the quality of each enrolled study. Publication bias was determined using funnel plots together with Egger’s tests. The Grading of Recommendations Assessment, Development, and Evaluation (GRADE) was used to evaluate the quality of the evidence.

**Results:**

Overall, we included 10,339 UTUC patients from twenty-five retrospective studies. The results indicated that elevated preoperative NLR, PLR, and MLR were significantly associated with worse OS, CSS, DFS/RFS/MFS, and PFS in the UTUC patients undergoing RNU. Furthermore, the results of sensitivity and subgroup analyses demonstrated the rationality and reliability of the results.

**Conclusions:**

The present meta-analysis demonstrated a significant association between elevated preoperative NLR, PLR, and MLR and poor prognosis in patients with surgically treated UTUC. Hence, lymphocyte-related systemic inflammatory biomarkers, in conjunction with clinicopathological factors, molecular markers, and other prognostic indicators, could be helpful to determine the primary treatment strategies and to design individualized follow-up plans for UTUC patients.

**Supplementary Information:**

The online version contains supplementary material available at 10.1186/s12957-020-02048-7.

## Background

Upper tract urothelial carcinoma (UTUC) is a relatively uncommon malignancy, accounting for only 5 to 10% of all urothelial carcinomas. It has an estimated annual incidence of almost 1 to 2 cases per 100,000 individuals [1, 2]. Despite its rareness, UTUC is an invasive and malignant disease with a large proportion of high-grade and locally advanced tumors at diagnosis [3]. Currently, radical nephroureterectomy (RNU) with bladder cuff excision is the standard primary treatment strategy for high-risk UTUC patients [4]. Nevertheless, some patients experience disease recurrence and progression, especially those with lymph-node involvement or advanced disease [5].

Current prognostic models are based on preoperative factors, such as tumor stage, grade (on biopsy and cytology), tumor location, and postoperative predictors like pathological T stage, lymph node involvement, and lymphovascular invasion [4, 6]. At the stage of designing individualized treatment strategies, the patients at low-risk and high-risk UTUC are stratified according to the preoperative factors to identify those that are more suitable for kidney-sparing surgery, radical extirpative treatment, or targeted systemic therapies. Moreover, after the primary treatment, those prognostic factors would be helpful to predict the clinical outcomes of the UTUC patients, allowing the clinicians to plan rigorous surveillance protocols or to determine the need for adjuvant chemotherapy. However, the current prognostic system has low accuracy that limited its clinical applications in UTUC patients [7]. Therefore, in this sense, preoperative lymphocyte-related systemic inflammatory biomarkers may improve the accuracy of current prognostic models in the risk stratification and the outcome prediction in UTUC patients who underwent RNU.

Tumor-associated inflammation is an important factor in the development of malignancies and promotes all stages of tumorigenesis [8, 9]. Additionally, the host immune response to malignancy might lead to changes in the levels of lymphocytes, neutrophils, platelets, and monocytes. Studies have reported several lymphocyte-related systemic inflammatory biomarkers, such as neutrophil-to-lymphocyte ratio (NLR), platelet-to-lymphocyte ratio (PLR), and monocyte-to-lymphocyte ratio (MLR), to have prognostic roles in a series of malignancies [10–12]. Although the predictive values of preoperative NLR, PLR, and MLR in UTUC patients have been demonstrated in several studies [13–20], these studies are limited by sample size and lack of standardized outcome definitions. Therefore, the aim of this meta-analysis was to elucidate the prognostic values of preoperative NLR, PLR, and MLR in UTUC patients treated with RNU.

## Methods

The present systematic review and meta-analysis was pre-registered in PROSPERO (CRD42020186531) and performed according to the Preferred Reporting Items for Systematic Reviews and Meta-analysis (PRISMA) guidelines [21].

### Search strategy

We performed a comprehensive search of the PubMed, EMBASE, Web of Science, and Cochrane Library databases for the literature, using a combination of the following terms: “upper tract urothelial carcinoma”, “upper urinary tract cancer”, “UTUC”, “prognosis”, “prognostic factors”, and “prognoses” from inception of the database to April 2020 (Additional file 1). Two investigators independently performed the literature search and resolved any disagreements via discussion.

### Inclusion and exclusion criteria

Study eligibility was determined using the PICOS (Population, Intervention, Comparator, Outcome, and Study design) approach. Prospective or retrospective cohort studies were considered eligible if they investigated UTUC patients having high NLR, PLR, and MLR (P) before RNU treatment (I) compared with patients having low NLR, PLR, and MLR (C) to assess the independent predictor of overall survival (OS), cancer-specific survival (CSS), disease-free survival (DFS), recurrence-free survival (RFS), metastasis-free survival (MFS), and progression-free survival (PFS) (O) using multivariate Cox regression analysis (S). The exclusion criteria were as follows: (1) reviews, case reports, conference abstracts, letters, and editorials; (2) studies without sufficient data; and (3) duplicate publications. Any disagreement was settled via discussion with a third investigator.

### Data extraction

Two investigators individually extracted the following items from each eligible study: name of the first author, publication year, recruitment region, study design, inclusion period, number of patients, gender, age, treatment, cutoff value, follow-up duration, and survival outcomes expressed as hazard ratios (HRs) for OS, CSS, DFS, RFS, MFS, and PFS with their 95% confidence intervals (CIs) from the multivariate analysis. Disagreements were resolved by consensus between the two investigators.

### Quality assessment

The Newcastle-Ottawa Scale (NOS) was applied to assess the quality of each enrolled study [22] and includes three factors: selection, comparability, and exposure. The total score ranged from 0 to 9, and the score of 3 or less, 4–6, or 7 or more were considered to have low, intermediate, or high quality, respectively.

### Statistical analysis

The endpoints of the present meta-analysis were OS, CSS, DFS, RFS, MFS, and PFS in UTUC patients treated with RNU. We extracted and combined HRs with the corresponding 95% CIs from every eligible study to analyze the prognostic value of NLR, PLR, and MLR. Heterogeneity between the studies was evaluated by Cochran’s Q test and *I*^2^ statistic. A random effects model was applied to calculate the pooled HRs and 95% CIs if there was significant heterogeneity among the enrolled studies (*I*^2^ > 50% or *P* < 0.10); otherwise, the fixed effects model was adopted (*I*^2^ < 50% or *P* > 0.10). In addition, sensitivity analyses were performed by sequentially excluding each study to assess the stability of the results. Subgroup analyses, stratified by different study features, were conducted to evaluate the potential factors contributing to heterogeneity. The presence of publication bias was evaluated using both the funnel plot and Egger’s test. Statistical analyses were carried out with the Stata 12.0 and Review Manager 5.3 software. A value of *P* < 0.05 was considered statistically significant.

### Quality of evidence

The Grading of Recommendations Assessment, Development, and Evaluation (GRADE) was used to assess the quality of the evidence of prognostic values of preoperative NLR, PLR, and MLR in the UTUC patients treated with RNU [23].

## Results

### Study selection

**Fig. 1:**
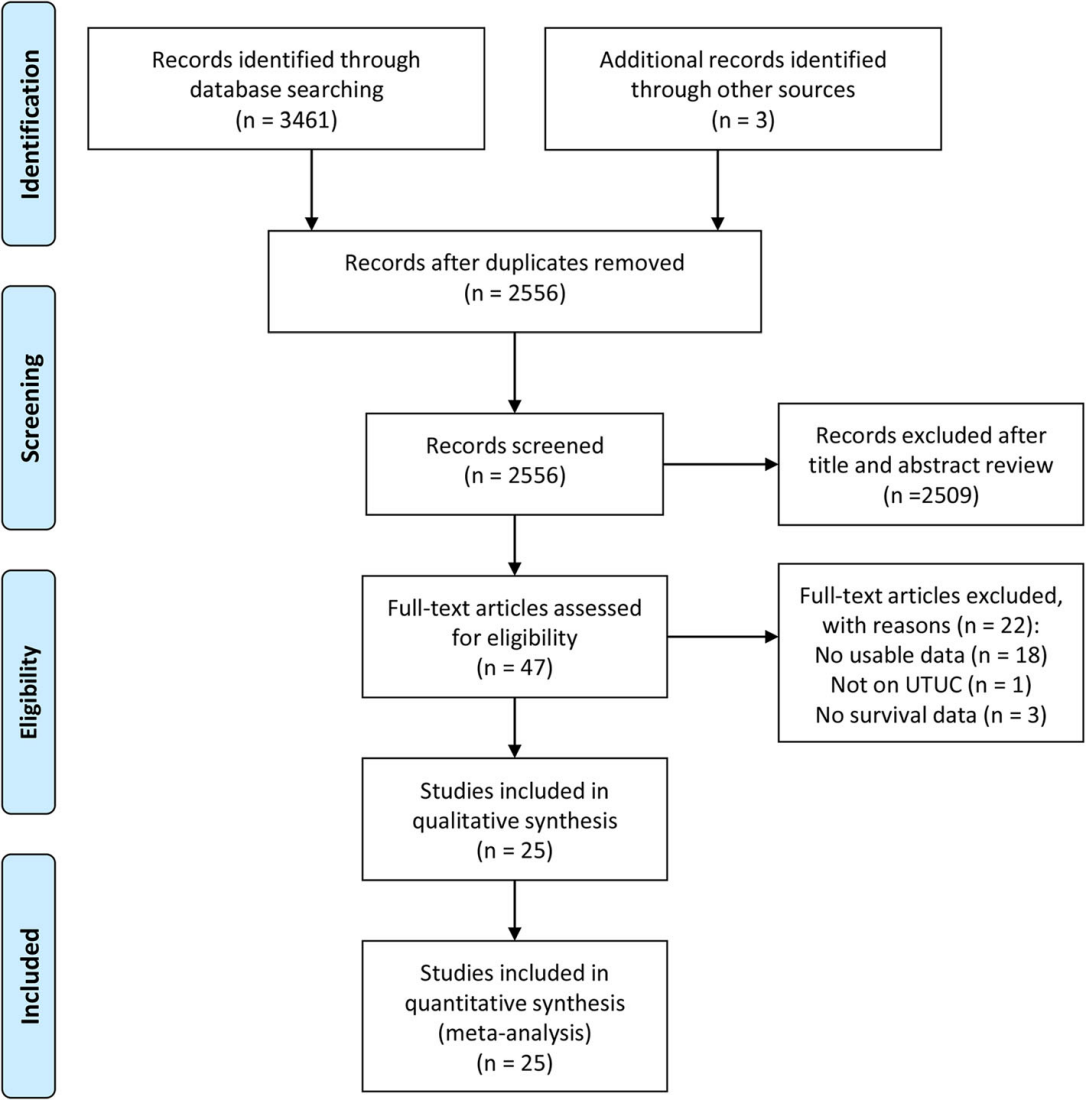
The flowchart indicated the process of study selection according to the Preferred Reporting Items for Systematic Reviews and Meta-Analyses guidelines (PRISMA)

In our database search, we identified 3461 potentially relevant studies. Particularly, we recognized three studies from the reference lists of these relevant studies. After the removal of duplicates, we viewed the titles and abstracts of the remaining 2556 articles. Subsequently, we assessed the full-text of 47 articles. Finally, we included 25 retrospective cohort studies in the present meta-analysis [13–20, 24–40]. Figure 1 presents the study selection process as a flowchart.

### Study characteristics

Table 1 summarizes the main characteristics of the included studies. Overall, 10,339 UTUC cases were included with 6685 males and 3654 females. All 25 studies were retrospective in design and published between 2013 and 2020. Among them, there were twenty [13–16, 18–20, 24, 26–29, 31, 33, 34, 36–40], nine [13–17, 28, 32–34], and six [13, 15, 16, 25, 30, 34] studies evaluating the relationship between the proposed predictors (NLR, PLR, and MLR) and clinical outcomes, respectively. The median age ranged from 62 to 71 years, and the median follow-ups ranged from 28 to 60.9 months. The NOS scores ranged from 6 to 8, indicating a moderate to high quality of the included studies (Additional file 2).

**Table 1:** Characteristics of the studies included in the meta-analysis

Author and year	Period	Region	Sample size	Gender (M/F)	Median age	Type of biomarker	Cutoff value			Surgery	NAC	AC	Outcome	Follow-up (months)	NOS score
							NLR	PLR	MLR						
Xu et al. 2020 [24]	2003–2016	China	703	399/304	67	NLR	2.5	-	-	RNU	0	287	OS, CSS, RFS	42 (1–168)	7
Jan et al. 2019 [13]	2007–2017	Taiwan	424	189/235	70	NLR, PLR, MLR	4.0	150	0.4	RNU	0	40	OS, CSS, PFS	35 (14–60)	7
Kuroda et al. 2019 [14]	1999–2017	Japan	187	138/49	71	NLR, PLR	2.035	165	-	RNU	4	53	CSS, RFS	Mean = 49.2 (3.4-209.2)	7
Li et al. 2019 [25]	2008–2017	China	704	401/303	Mean = 66	MLR	-	-	3.6	RNU	0	286	OS, CSS, RFS	36 (34–43)	7
Zheng et al. 2019 [15]	2005–2015	China	259	185/74	Mean = 67.5	NLR, PLR, MLR	2.53	126.88	0.35	RNU	0	28	OS, CSS, MFS	33.3 (15.5–64.2)	8
Kohada et al. 2018 [26]	1999–2016	Japan	148	112/36	Mean = 71	NLR	3.0	-	-	RNU	0	25	CSS, RFS	35.5	7
Nishikawa et al. 2018 [27]	2005–2015	Japan	135	106/29	69	NLR	3.0	-	-	RNU	0	30	RFS	36.1	7
Son et al. 2018 [28]	2004–2015	Korea	1137	825/312	69	NLR, PLR	3.3	142	-	RNU	NA	348	CSS, RFS	39.1 (18.3–63.8)	7
Tan et al. 2018 [29]	2004–2015	China	717	408/309	67	NLR	2.5	-	-	RNU	0	291	OS, CSS, RFS	42 (1–167)	7
Zhang et al. 2018 [30]	1990–2011	China	100	79/21	Mean = 68.3	MLR	-	-	3.0	RNU	0	NA	OS	Mean = 45.83 (1–151)	7
Altan et al. 2017 [16]	1990–2017	Turkey	113	86/27	Mean = 63.7	NLR, PLR, MLR	2.9	150	2.9	RNU	NA	0	DFS, PFS	34 (3–186)	6
Dalpiaz et al. 2017 [17]	1990–2012	Austria	180	109/71	70	PLR	-	150	-	RNU	NA	NA	OS, CSS	30	7
Huang et al. 2017 [31]	2003–2013	China	425	279/146	Mean = 65.9	NLR	2.955	-	-	RNU	0	86	OS, CSS	38.5 (23–62)	7
Jiang et al. 2017 [32]	2005-2015	China	113	76/37	Mean = 64.86	PLR	-	150	-	RNU	NA	32	CSS, RFS	29 (2–113)	6
Kang et al. 2017 [18]	1994–2012	Korea	90	67/23	62	NLR	2.9	-	-	RNU	0	90	OS, CSS	NA	6
Vartolomei et al. 2017 [19]	1990–2008	Austria	2274	1527/747	69	NLR	2.7	-	-	RNU	0	217	CSS, RFS	40 (20–76)	8
Cheng et al. 2016 [20]	2005–2010	Taiwan	195	79/116	Mean = 68	NLR	2.7	-	-	RNU	NA	35	OS, CSS	36	7
Huang et al. 2016 [33]	2002–2013	China	481	311/170	Mean = 65.8	NLR, PLR	3.22	241.2	-	RNU	0	96	OS, CSS	40 (24–64)	7
Song et al. 2016 [34]	2005–2011	China	140	86/54	67	NLR, PLR, MLR	2.2	128	3.6	RNU	NA	0	DFS, PFS	45 (11–108)	7
Hutterer et al. 2015 [35]	1990–2012	Austria	182	111/71	Mean = 69.0	MLR	-	-	2.0	RNU	NA	NA	OS	NA	7
Tanaka et al. 2015 [36]	1995–2011	Japan	394	289/105	70	NLR	3.0	-	-	RNU	0	88	CSS, RFS	30 (15–63)	7
Dalpiaz et al. 2014 [37]	1990–2012	Austria	202	122/80	Mean = 69.3	NLR	2.7	-	-	RNU	0	0	OS, CSS	45 (0–199)	8
Luo et al. 2014 [38]	2005–2010	Taiwan	234	102/132	NA	NLR	3.0	-	-	RNU	0	0	CSS, MFS	Mean = 40.7	7
Tanaka et al. 2014 [39]	1993–2011	Japan	665	493/172	Mean = 70	NLR	3.0	-	-	RNU	0	129	CSS, RFS	28 (14–57)	7
Azuma et al. 2013 [40]	1994–2008	Japan	137	106/31	Mean = 69.4	NLR	2.5	-	-	RNU	NA	NA	CSS, RFS	60.9 (6.9–187.3)	7

*AC* Adjuvant chemotherapy, *NA* Not available, *NAC* Neoadjuvant chemotherapy, *NOS score* Newcastle-Ottawa Scale score, *RNU* Radical nephroureterectomy

### Prognostic value of NLR in UTUC patients

Twenty studies with 9060 patients had evaluated the prognostic value of NLR in the UTUC patients [13–16, 18–20, 24, 26–29, 31, 33, 34, 36–40].

**Fig. 2:**
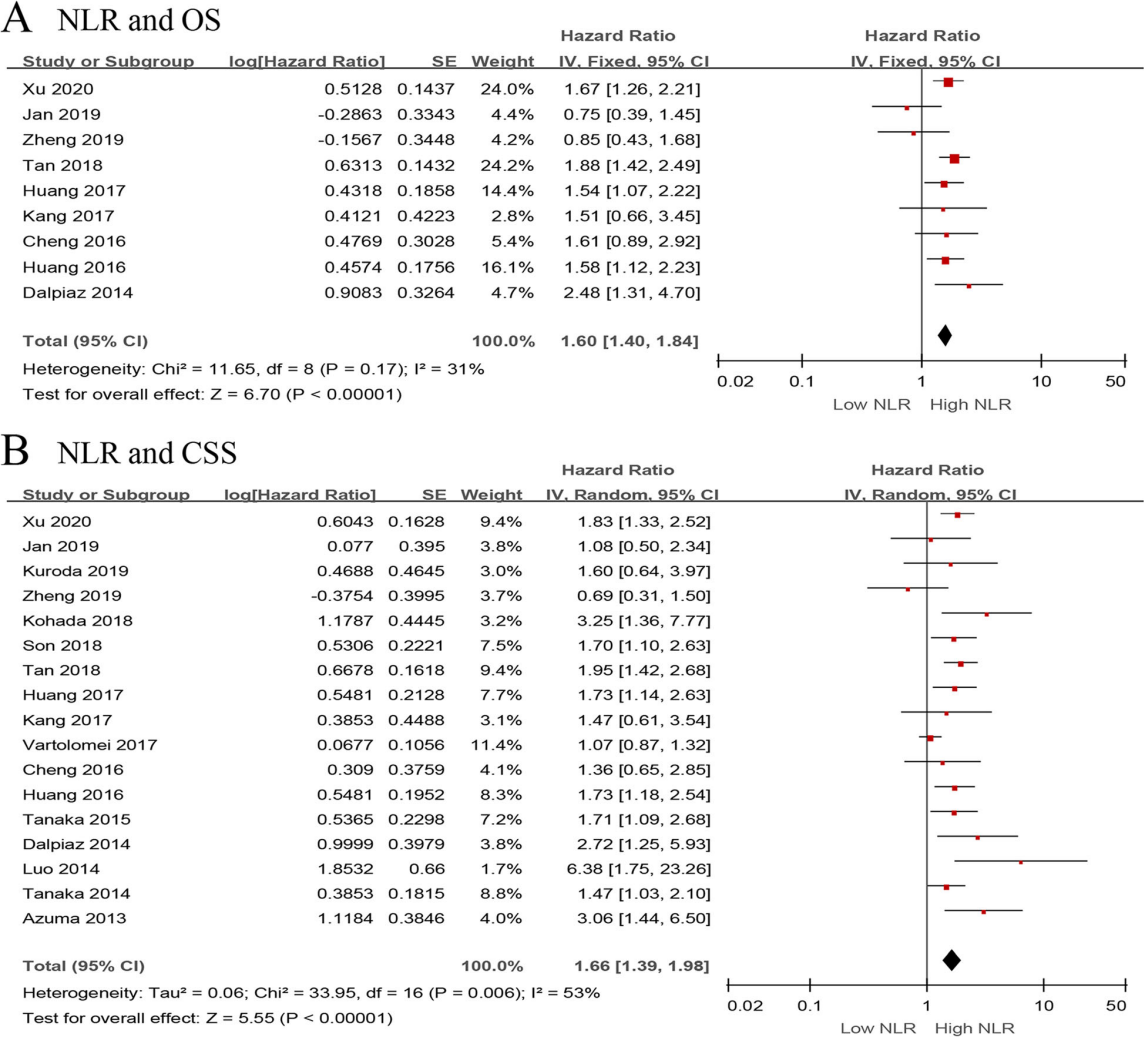
Forest plots of included studies evaluating the association between **a** NLR and OS and **b** NLR and CSS in UTUC patients treated with RNU

We investigated the role of preoperative NLR as a predictor of OS using nine studies with 3496 UTUC patients [13, 15, 18, 20, 24, 29, 31, 33, 37]. The synthesized analysis showed that elevated preoperative NLR significantly correlated with shorter OS in the UTUC patients (pooled HR 1.60, 95% CI 1.40–1.84, *P* < 0.001, *I*^2^ = 31%; Fig. 2a). The results of the sensitivity analysis showed that heterogeneity markedly reduced (*I*^2^ = 0%, *P* = 0.51) after excluding Jan et al.’s study [13] (Additional file 3). Furthermore, the funnel plot identified it over the pseudo 95% CI (Fig. 4a). By excluding this study, the recalculated result suggested that a significant association between the preoperative NLR and OS without heterogeneity (pooled HR 1.66, 95% CI 1.44–1.91, *P* < 0.001, *I*^2^ = 0%; Additional file 4).

Seventeen studies, comprising 8672 UTUC patients, reported the data of NLR and CSS [13–15, 18–20, 24, 26, 28, 29, 31, 33, 36–40]. The pooled results, based on random effects model, indicated that elevated preoperative NLR significantly associated with shorter CSS in the UTUC patients (pooled HR 1.66, 95% CI 1.39–1.98, *P* < 0.001, *I*^2^ = 53%; Fig. 2b). The sensitivity analysis showed that heterogeneity was evidently reduced (*I*^2^ = 18%, *P* = 0.25) after excluding Vartolomei et al.’s study [19]. The pooled HR, recalculated using the fixed effects model, was 1.75 (95% CI 1.54–1.98, *P* < 0.001; Additional file 3). In addition, the funnel plot identified three studies over the pseudo 95% CI [15, 19, 20] (Fig. 4b). By excluding these studies, the recalculated results demonstrated a significant association between the preoperative NLR and CSS without heterogeneity (pooled HR 1.81, 95% CI 1.59–2.05, *P* < 0.001, *I*^2^ = 0%; Additional file 4).

**Fig. 3:**
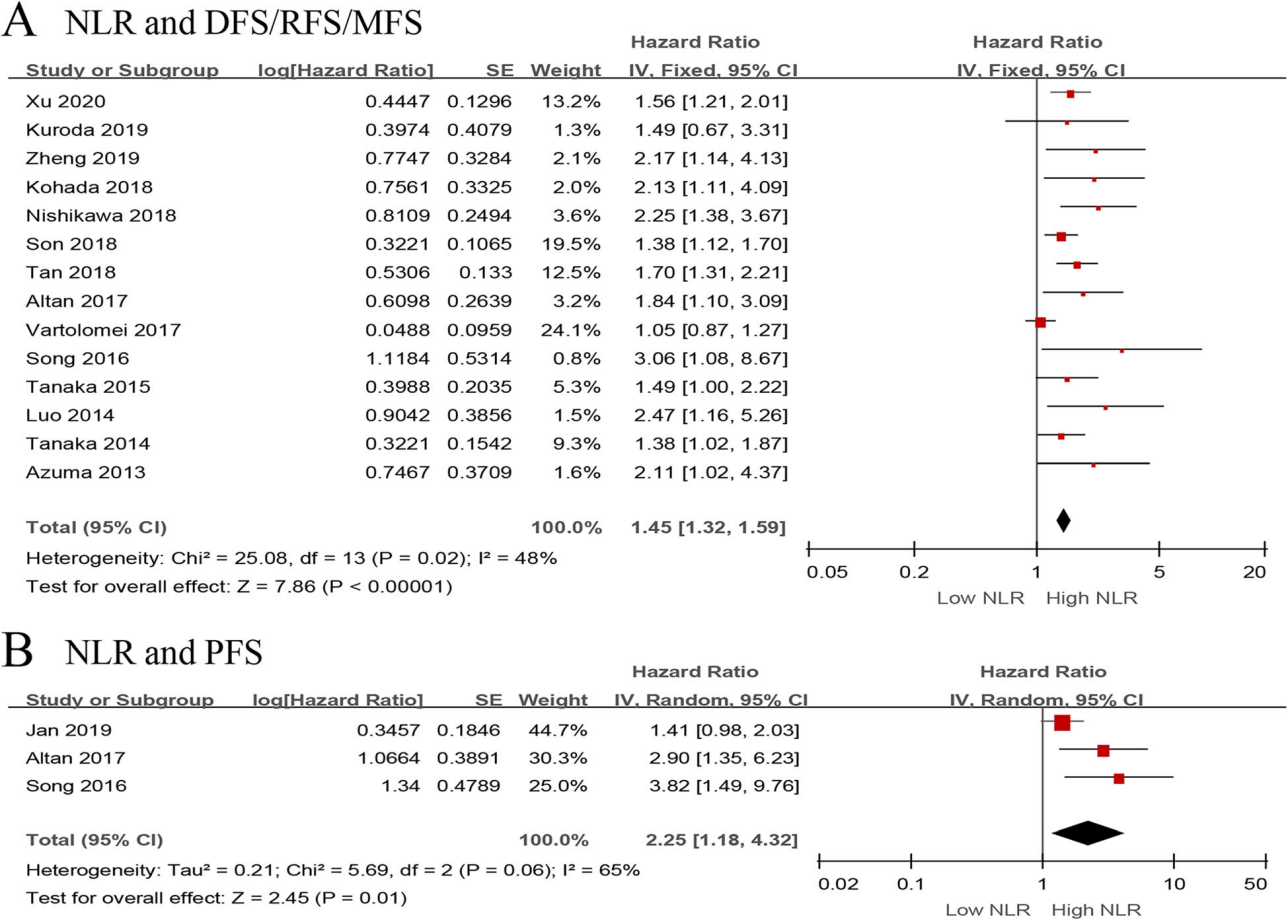
Forest plots of included studies evaluating the association between **a** NLR and DFS/RFS/MFS and **b** NLR and PFS in UTUC patients treated with RNU

**Fig. 4:**
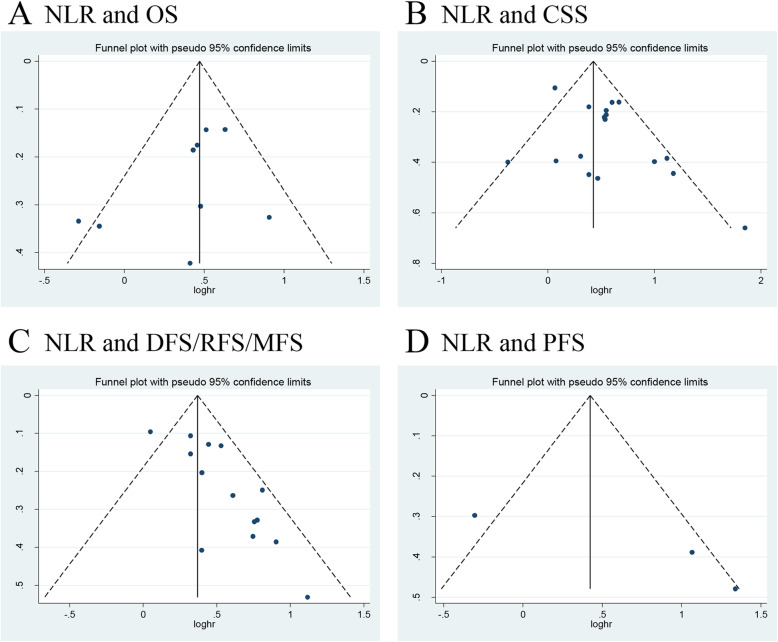
Funnel plots of **a** NLR and OS, **b** NLR and CSS, **c** NLR and DFS/RFS/MFS, and **d** NLR and PFS

Next, we evaluated the relationship between NLR and DFS/RFS/MFS in the UTUC patients using fourteen studies with 7243 patients [14–16, 19, 24, 26–29, 34, 36, 38–40]. The forest plot revealed that the elevated NLR significantly associated with worse DFS/RFS/MFS (pooled HR 1.45, 95% CI 1.32–1.59, *P* < 0.001, *I*^2^ = 48%; Fig. 3a). The results of the sensitivity analysis showed that heterogeneity was distinctly reduced (*I*^2^ = 0%, *P* = 0.59) after removing Vartolomei et al.’s study [19] (Additional file 3). Moreover, the funnel plot identified it over the pseudo 95% CI (Fig. 4c). By excluding this study, the recalculated result indicated a significant association between the preoperative NLR and DFS/RFS/MFS without heterogeneity (pooled HR 1.60, 95% CI 1.44–1.78, *P* < 0.001, *I*^2^ = 0%; Additional file 4).

Only three studies, including 677 patients with UTUC, evaluated the association of NLR with PFS [13, 16, 34]. The meta-analysis results showed that elevated preoperative NLR significantly associated with worse PFS (pooled HR 2.25, 95% CI 1.18–4.32, *P* = 0.01, *I*^2^ = 65%; Fig. 3b). The sensitivity analysis showed that heterogeneity was evidently reduced (*I*^2^ = 0%, *P* = 0.66) after excluding Jan et al.’s study [13] (Additional file 3). Additionally, the funnel plot identified it over the pseudo 95% CI (Fig. 4d). By excluding this study, the recalculated result suggested a significant association between the preoperative NLR and PFS without heterogeneity (pooled HR 3.24, 95% CI 1.79–5.85, *P* < 0.001, *I*^2^ = 0%; Additional file 4).

### Prognostic value of PLR in UTUC patients

Nine studies comprising of 3034 patients evaluated the prognostic value of PLR in the UTUC patients [13–17, 28, 32–34].

**Fig. 5:**
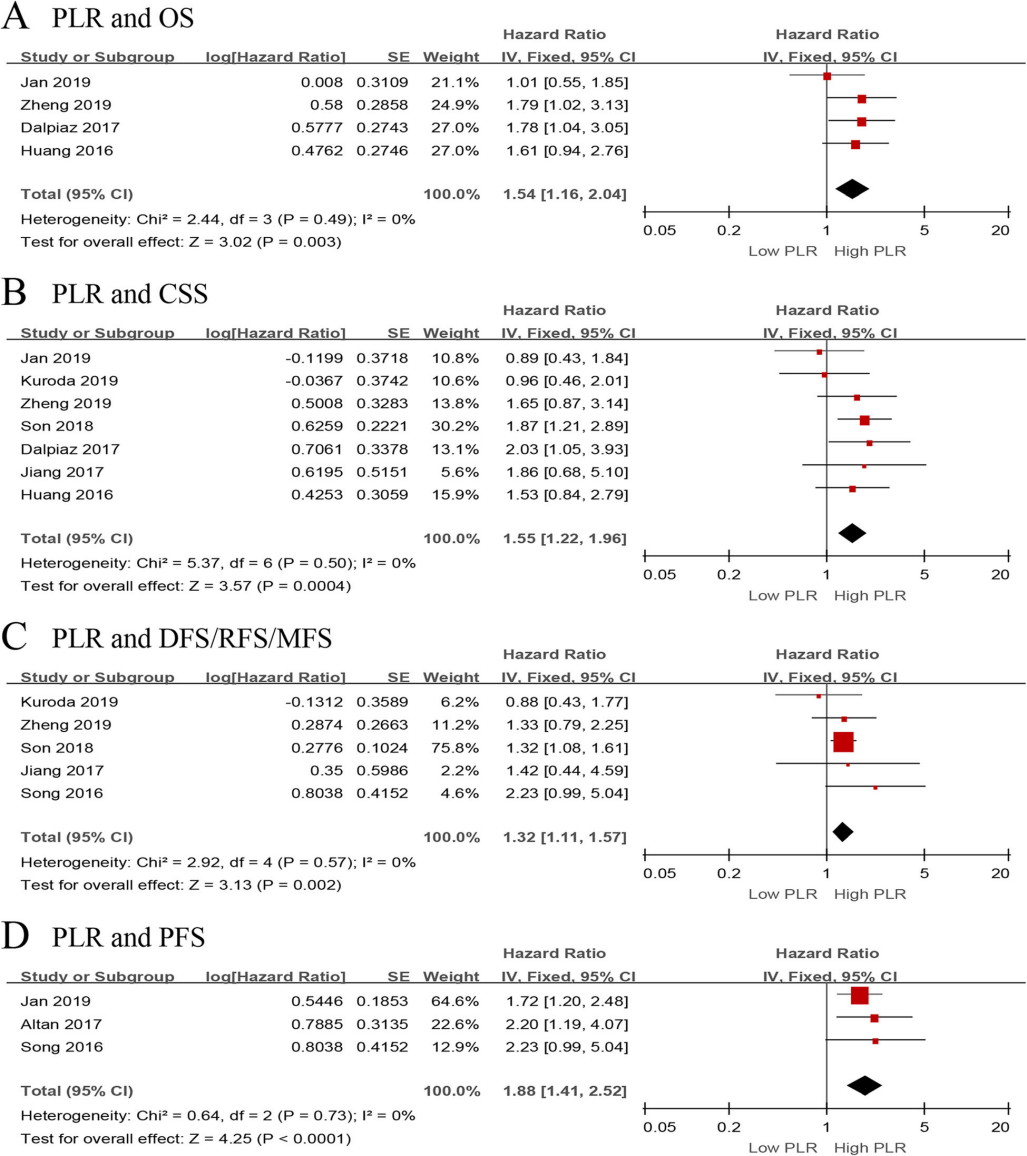
Forest plots of included studies evaluating the association between **a** PLR and OS, **b** PLR and CSS, **c** PLR and DFS/RFS/MFS, and **d** PLR and PFS in UTUC patients treated with RNU

**Fig. 6:**
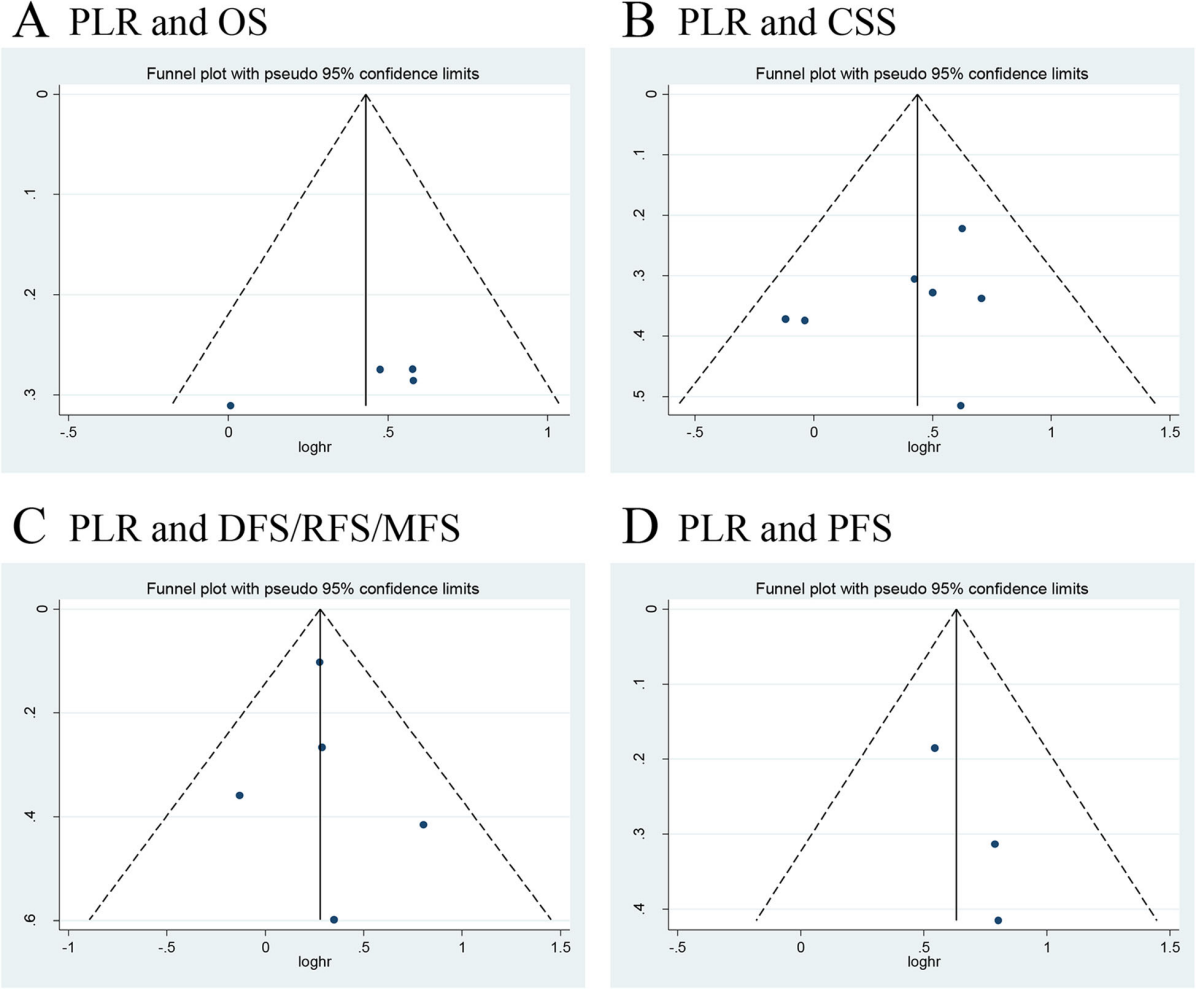
Funnel plots of **a** PLR and OS, **b** PLR and CSS, **c** PLR and DFS/RFS/MFS, and **d** PLR and PFS

We investigated the role of preoperative PLR as a predictor of OS using four studies with 1344 patients [13, 15, 17, 33]. The synthesized analysis showed that elevated preoperative PLR significantly correlated with shorter OS in the UTUC patients (pooled HR 1.54, 95% CI 1.16–2.04, *P* = 0.003, *I*^2^ = 0%; Fig. 5a). Furthermore, the sensitivity analysis did not find any study that significantly affected heterogeneity (Additional file 5), and the funnel plot did not identify any specific study over the pseudo 95% CI (Fig. 6a).

Seven studies, comprising 2781 UTUC patients, reported the data of PLR and CSS [13–15, 17, 28, 32, 33]. The pooled results, based on the fixed effects model, indicated that elevated preoperative PLR significantly associated with shorter CSS in UTUC (pooled HR 1.55, 95% CI 1.22–1.96, *P* < 0.001, *I*^2^ = 0%; Fig. 5b). In addition, the sensitivity analysis did not find any study that significantly affected heterogeneity (Additional file 5), and the funnel plot did not identify any study over the pseudo 95% CI (Fig. 6b).

Next, we evaluated the relationship between the PLR value and DFS/RFS/MFS using five studies with 1836 UTUC patients [14, 15, 28, 32, 34]. The forest plot revealed that elevated PLR significantly associated with worse DFS/RFS/MFS (pooled HR 1.32, 95% CI 1.11–1.57, *P* = 0.002, *I*^2^ = 0%; Fig. 5c). Moreover, the sensitivity analysis did not find a single study that significantly affected heterogeneity (Additional file 5), and the funnel plot did not identify study over the pseudo 95% CI (Fig. 6c).

Only three studies, including 677 patients with UTUC, evaluated the association of PLR with PFS [13, 16, 34]. The meta-analysis revealed that elevated preoperative PLR significantly associated with worse PFS (pooled HR 1.88, 95% CI 1.41–2.52, *P* < 0.001, *I*^2^ = 0%; Fig. 5d). Additionally, the sensitivity analysis did not find any study that significantly affected heterogeneity (Additional file 5), and the funnel plot did not identify any study over the pseudo 95% CI (Fig. 6d).

### Prognostic value of MLR in UTUC patients

Six studies, including 1740 patients, evaluated the prognostic value of MLR in the UTUC patients [13, 15, 16, 25, 30, 34].

**Fig. 7:**
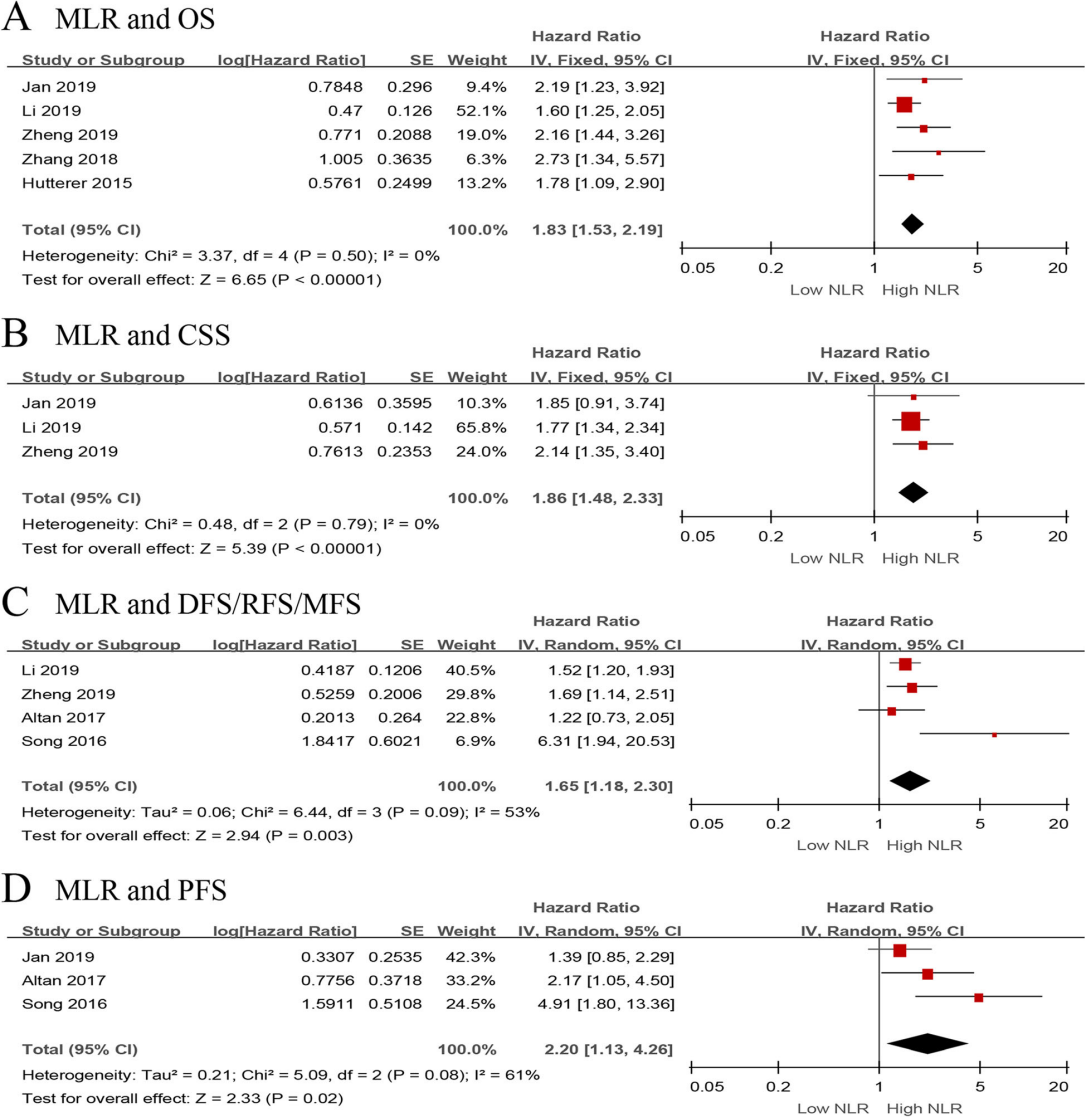
Forest plots of included studies evaluating the association between **a** MLR and OS, **b** MLR and CSS, **c** MLR and DFS/RFS/MFS, and **d** MLR and PFS in UTUC patients treated with RNU

**Fig. 8:**
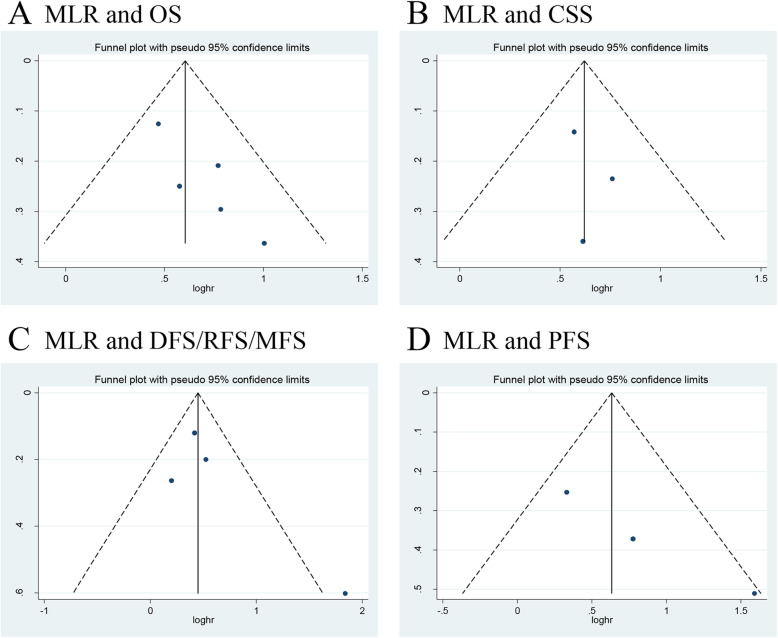
Funnel plots of **a** MLR and OS, **b** MLR and CSS, **c** MLR and DFS/RFS/MFS, and **d** MLR and PFS

We investigated the role of preoperative MLR as a predictor of OS in five studies with 1669 UTUC patients [13, 15, 25, 30, 35]. The synthesized analysis showed that elevated preoperative MLR significantly correlated with shorter OS in the UTUC patients (pooled HR 1.83, 95% CI 1.53–2.19, *P* < 0.001, *I*^2^ = 0%; Fig. 7a). Furthermore, the sensitivity analysis did not find any study that significantly affected heterogeneity (Additional file 6), and the funnel plot did not identify any study over the pseudo 95% CI (Fig. 8a).

Three studies, comprising 1387 UTUC patients, reported the data of MLR and CSS [13, 15, 25]. The pooled results, based on the fixed effects model, indicated that elevated preoperative MLR significantly associated with shorter CSS in the UTUC patients (pooled HR 1.86, 95% CI 1.48–2.33, *P* < 0.001, *I*^2^ = 0%; Fig. 7b). In addition, the sensitivity analysis did not find a single study that significantly affected heterogeneity (Additional file 6), and the funnel plot did not identify any study over the pseudo 95% CI (Fig. 8b).

Next, we evaluated the relationship between MLR and DFS/RFS/MFS outcomes in UTUC patients. We included four studies with 1216 patients [15, 16, 25, 34]. The forest plot revealed that elevated MLR showed a significant association with worse outcome for DFS/RFS/MFS (pooled HR 1.65, 95% CI 1.18–2.30, *P* = 0.003, *I*^2^ = 53%; Fig. 7c). The sensitivity analysis showed that heterogeneity was evidently reduced (*I*^2^ = 0%, *P* = 0.62) after excluding Song et al.’s study [34] (Additional file 6). Moreover, the funnel plot identified it over the pseudo 95% CI (Fig. 8c). By excluding this study, the recalculated result revealed a significant association between preoperative MLR and DFS/RFS/MFS without heterogeneity (pooled HR 1.51, 95% CI 1.25–1.83, *P* < 0.001, *I*^2^ = 0%; Additional file 7).

Only three studies, including 677 patients with UTUC, evaluated the association of MLR with PFS [13, 16, 34]. The meta-analysis showed that elevated preoperative MLR significantly associated with a worse outcome for PFS (pooled HR 2.20, 95% CI 1.13–4.26, *P* = 0.02, *I*^2^ = 61%; Fig. 7d). The sensitivity analysis indicated that heterogeneity was distinctly reduced (*I*^2^ = 0%, *P* = 0.32) after excluding Song et al.’s study [34]. The pooled HR, recalculated using the fixed effects model, was 1.60 (95% CI 1.06–2.42, *P* = 0.02; Additional file 6). Additionally, the funnel plot did not identify any study over the pseudo 95% CI (Fig. 8d).

### Subgroup analysis

Our results confirmed that elevated pretreatment NLR could be a predictor of CSS based on 17 studies. However, heterogeneity remained relatively significant after the sensitivity analysis. As different study features were involved, we further carried out a subgroup analysis to explore the source of this heterogeneity (Table 2). In the subgroup analysis based on research regions, elevated pretreatment NLR was significantly associated with worse CSS in studies performed in Asia, whereas no significant association was observed in studies conducted in Europe; the latter could be due to the limited number of investigations. Interestingly, when stratified by the cutoff value of NLR, heterogeneity was evidently reduced, and the results showed that NLR significantly associated with CSS in both the cutoff value of NLR > 2.70 and cutoff value ≤ 2.70 subgroups. Collectively, the results of subgroup analyses indicated that the median age, sample size, median follow-up months, and quality of study did not affect the relationship between NLR and CSS, whereas the sample size, median age, and cutoff value could be the potential source of heterogeneity.

**Table 2:** Subgroup analysis for NLR in UTUC patients

Subgroup	No. of studies	No. of patients	HR (95% CI)	*P* value	Heterogeneity		Effect model
					*I*^2^ (%)	Ph	
Overall	17	8672	1.66 (1.39–1.98)	< 0.001	53	0.006	Random
Region							
Europe	2	2476	1.58 (0.64–3.88)	0.32	80	0.02	Random
Asia	15	6196	1.73 (1.52–1.96)	< 0.001	17	0.26	Fixed
Sample size							
≥ 200	13	8110	1.58 (1.31–1.91)	< 0.001	57	0.006	Random
< 200	4	562	2.28 (1.49–3.28)	< 0.001	0	0.43	Fixed
Median age (years)							
≥ 70	5	1818	1.59 (1.25–2.03)	< 0.001	0	0.43	Fixed
< 70	11	6620	1.62 (1.30–2.01)	< 0.001	60	0.005	Random
Cutoff value of NLR							
> 2.70	6	1986	1.60 (1.26–2.02)	< 0.001	0	0.42	Fixed
≤ 2.70	11	6686	1.78 (1.53–2.07)	< 0.001	8	0.37	Fixed
Median time of follow-up (months)							
≥ 36	11	6692	1.77 (1.41–2.22)	< 0.001	61	0.004	Random
< 36	5	1890	1.47 (1.16–1.87)	0.002	49	0.10	Fixed
Quality of study							
High	16	8582	1.67 (1.39–2.01)	< 0.001	56	0.003	Random
Moderate	1	90	-	-	-	-	-

### Publication bias

We assessed the publication bias using the Egger’s tests. The results showed no significant publication bias in NLR (*P* = 0.209, 0.061, 0.201, and 0.207, respectively), PLR (*P* = 0.104, 0.368, 0.857, and 0.201, respectively), and MLR (*P* = 0.051, 0.641, 0.362, and 0.083, respectively) indicating the robustness of the results.

### Quality of evidence

We assessed the quality of evidence for OS, CSS, DFS/RFS/MFS, and PFS, which was critical in evaluating the prognosis of UTUC patients. As mentioned in Additional file 8, the quality of evidence was “low” due to observational studies and “very low” due to observational studies and high heterogeneity.

## Discussion

It is a known fact that tumor microenvironment and the cancer-associated inflammatory response play an important role in the development and progression of cancer. Several studies evaluated the lymphocyte-related systemic inflammatory biomarkers, including NLR, PLR, and MLR, as prognostic factors in UTUC but produced inconsistent findings. Thus, the aim of our systematic review and meta-analysis of 10,339 UTUC cases was to summarize and to analyze the current evidence regarding the predictive value of preoperative lymphocyte-related biomarkers. The results indicated that elevated preoperative NLR, PLR, and MLR significantly associated with the worse OS, CSS, DFS/RFS/MFS, and PFS. Subsequently, the sensitivity and subgroup analyses further demonstrated the reliability and rationality of our findings. Collectively, the pooled data from this meta-analysis confirmed that the preoperative NLR, PLR, and MLR could predict the clinical outcomes and may serve as reliable prognostic indicators in UTUC patients treated with RNU.

The application of prognostic biomarkers could enhance the risk stratification, help design individualized treatment, and determine the follow-up schedule. At the stage of customizing therapeutic strategies, NLR, PLR, and MLR, together with the clinicopathological factors and molecular markers, could be instrumental in risk stratification for the UTUC patients in a preoperative setting. For patients with low-risk UTUC, the clinicians could offer kidney-sparing surgery as the primary treatment option to protect kidney function and spare the morbidity associated with radical surgery. However, for patients with high-risk UTUC, urologists could perform lymph node dissection or intravesical chemotherapy on the basis of RNU to maximally improve the prognosis of patients. Furthermore, after the surgery, NLR, PLR, and MLR, in conjunction with other prognostic indicators, such as tumor grade, lymph node involvement, and surgical margins, could precisely predict the clinical outcomes of the UTUC patients. Therefore, the urologists could plan more frequent and stricter follow-up strategies for the patients with potentially poor prognosis. In summary, at the different stages of diagnostics, therapeutics, and follow-ups, the application of these preoperative lymphocyte-related systemic inflammatory biomarkers could potentially increase the precision of current prognostic models and could be helpful in making clinical decisions.

The exact mechanisms by which these biomarkers have prognostic value in UTUC patients remain unclear. The underlying mechanisms may be associated with the functions of neutrophils, platelets, monocytes, and lymphocytes. Neutrophils are essential effector cells in the acute phase of inflammation, playing a key role in the resistance against microbes [41]. Some studies have shown that neutrophils were involved in the inhibition of the anti-tumor immune surveillance and in the extracellular matrix remodeling, thus promoting the migration of cancer cells [42]. Besides, it has been confirmed that neutrophils create an inflammatory environment by producing tumor growth promoters, including the vascular endothelial growth factor and other immunoregulatory mediators, resulting in cancer angiogenesis and progression [41]. The platelets are considered critical components of hemostasis. However, some studies questioned this function and explored their role in cancer. A study reported that platelets could shield cancer cells from the cytotoxicity of the immune cells [9]. Additionally, the platelets could enhance the epithelial-mesenchymal transition of the tumor cells by directly contacting tumor cells or indirectly secreting prostaglandin E2 and platelet-derived growth factors [43]. Platelets may also play an important role in the generation of macrophages and neutrophils by recruiting and regulating the monocytic and granulocytic cells [44]. Some studies revealed that circulating monocytes could be recruited into the tumor microenvironment and polarized into tumor-associated macrophages, which are associated with worse survival [45]. Furthermore, macrophages derived from monocytes could enable cancer cells to evade immune destruction and promote aggressive invasion [46]. In contrast, the lymphocytes play a vital role in cell-mediated anti-tumor response. Tumor-infiltrating lymphocytes are common inflammatory cells in the tumor environment and are associated with prognosis and responsiveness to therapy in patients with solid tumors. Additionally, a decrease in peripheral lymphocytes could damage anti-tumor responses, resulting in tumor cell proliferation and metastasis [47]. Theoretically, NLR, PLR, and MLR not only represent the response to systemic inflammation in patients with malignancies, but also reflect the impaired cell-mediated immunity; thus, they could be considered as promising prognostic and predictive biomarkers in the UTUC patients who underwent RNU.

Collectively, our study thoroughly investigates the prognostic values of the preoperative lymphocyte-related systemic inflammatory biomarkers in patients with surgically treated UTUC. As effective prognostic biomarkers, NLR, PLR, and NLR have many advantages, including being economical, easily available, and simple to calculate. In this analysis, we found that elevated preoperative NLR, PLR, and MLR were associated with the recurrence of UTUC patients, especially intravesical recurrence after RNU. It was reported that recurrence in the bladder occurs in 22–47% of UTUC patients [4]. Therefore, it is imperative to perform bladder cuff excision on the basis of RNU and/or postoperative intravesical instillation after the surgery for the patients. This will help reduce the risk of tumor recurrence in the area of the distal ureter and its orifice. Moreover, urologists should perform more intense cystoscopy, urinary cytology, and computed tomography urography surveillance scheduling for patients with high-risk UTUC for early detection of bladder recurrence during the follow-ups. Notably, our study mainly investigated the prognostic value of the lymphocyte-related inflammatory biomarkers in UTUC patients treated with RNU. Although RNU was the standard primary treatment, some patients received neoadjuvant or adjuvant chemotherapy in the 25 studies that were included; this may have led to the observed heterogeneity. Therefore, better-designed studies are needed to assess the prognostic role of NLR, PLR, and MLR in UTUC patients receiving different treatments.

This study has several limitations. First, all the included studies were retrospective, with some of these being single-centered with small sample sizes. Second, the quality of evidence for the results of this study was “low” to “very low” according to the GRADE. Therefore, urologists should interpret our results cautiously when employing these biomarkers in daily clinical practice. Third, the optimal cutoff values of these biomarkers remain undetermined. Different cutoff thresholds might contribute to potential bias and heterogeneity. Fourth, this meta-analysis did not include the UTUC patients who received targeted therapies or immunotherapies. Thus, we require further investigations regarding the association between these biomarkers and the prognosis of metastatic UTUC patients.

## Conclusion

In summary, this meta-analysis demonstrated that elevated preoperative NLR, PLR, and MLR were associated with increased risks of mortality in UTUC patients. As non-invasive and easily accessible prognostic biomarkers, urologists could combine NLR, PLR, and MLR with clinicopathological factors, molecular markers, and other prognostic indicators to stratify the risk, individualize treatment strategies, and more precisely predict the clinical outcome for patients with surgically treated UTUC.

## Supplementary Information

**Additional file 1:.** Search strategy of present systematic review and meta-analysis.

**Additional file 2:.** Newcastle-Ottawa scale score of the reviewed studies.

**Additional file 3:.** Sensitivity analyses for preoperative NLR in UTUC patients treated with RNU.

**Additional file 4:.** Forest plots of the association between NLR and OS, CSS, DFS/RFS/MFS, and PFS after removing the three studies over the pseudo 95% CI according to funnel plot.

**Additional file 5:.** Sensitivity analyses for preoperative PLR in UTUC patients treated with RNU.

**Additional file 6:.** Sensitivity analyses for preoperative MLR in UTUC patients treated with RNU.

**Additional file 7:.** Forest plots of the association between MLR and DFS/RFS/MFS after removing the three studies over the pseudo 95% CI according to funnel plot.

**Additional file 8:.** Evaluation of the quality of evidence according to GRADE system.

## Data Availability

The data that support the findings of this study are available from the corresponding author upon reasonable request.

## Abbreviations

CI: Confidence intervals
CSS: Cancer-specific survival
DFS: Disease-free survival
GRADE: Grading of Recommendations Assessment, Development, and Evaluation
HR: Hazard ratio
MFS: Metastasis-free survival
MLR: Monocyte-to-lymphocyte ratio
NLR: Neutrophil-to-lymphocyte ratio
NOS: Newcastle-Ottawa Scale
OS: Overall survival
PFS: Progression-free survival
PLR: Platelet-to-lymphocyte ratio
PRISMA: Preferred Reporting Items for Systematic Reviews and Meta-analyses
RFS: Recurrence-free survival
RNU: Radical nephroureterectomy
UCs: Urothelial carcinomas
UTUC: Upper tract urothelial carcinoma
