# Supplementary material for: Prognostic value of preoperative lymphocyte-related systemic inflammatory biomarkers in upper tract urothelial carcinoma patients treated with radical nephroureterectomy: a systematic review and meta-analysis
Source: World J Surg Oncol. 2020 Oct 23;18:273. doi: 10.1186/s12957-020-02048-7 (PMC7585317; doi:10.1186/s12957-020-02048-7)
Supplement: Supplementary file 1 — Additional file 1:. Search strategy of present systematic review and meta-analysis. [file 12957_2020_2048_MOESM1_ESM.docx]

**Search strategy**

**PubMed**

((upper tract urothelial carcinoma) OR (upper urinary tract cancer) OR (UTUC)) AND ((prognosis) OR (prognostic factor) OR (prognoses))

**Web of science**

ALL=(“upper tract urothelial carcinoma” OR “upper urinary tract cancer” OR “UTUC”) AND ALL=(“prognosis” OR “prognostic factor” OR “prognoses”)

**Chochrane:**

#1 ((upper tract urothelial carcinoma) OR (upper urinary tract cancer) OR (UTUC))

#2 ((prognosis) OR (prognostic factor) OR (prognoses))

#3 #1 and #2

**Embase(Ovid SP):**

(“upper tract urothelial carcinoma” OR “upper urinary tract cancer” OR “UTUC”) AND (“prognosis” OR “prognostic factor” OR “prognoses”)
