# Supplementary material for: Prognostic value of preoperative lymphocyte-related systemic inflammatory biomarkers in upper tract urothelial carcinoma patients treated with radical nephroureterectomy: a systematic review and meta-analysis
Source: World J Surg Oncol. 2020 Oct 23;18:273. doi: 10.1186/s12957-020-02048-7 (PMC7585317; doi:10.1186/s12957-020-02048-7)
Supplement: Supplementary file 2 — Additional file 2:. Newcastle-Ottawa scale score of the reviewed studies. [file 12957_2020_2048_MOESM2_ESM.docx]

| Table S1 Newcastle-Ottawa scale score of the reviewed studies | | | | | | | | | |
| --- | --- | --- | --- | --- | --- | --- | --- | --- | --- |
| Study | Selection (4 stars) | | | | Comparability  (2 stars) | Outcome (3 stars) | | | Total  score |
|  | Representativeness of the exposed cohort | Selection of the non exposed cohort | Ascertainment  of exposure | Demonstration that outcome of interest was not present at start of study |  | Assessment of outcome | Was follow up long enough for outcomes to occur? | Adequacy of follow up of cohort |  |
| Xu [24] | ★ | ★ | ★ | ★ | - | ★ | ★ | ★ | 7 |
| Jan [13] | ★ | ★ | ★ | ★ | - | ★ | ★ | ★ | 7 |
| Kuroda [14] | ★ | ★ | ★ | ★ | - | ★ | ★ | ★ | 7 |
| Li [25] | ★ | ★ | ★ | ★ | - | ★ | ★ | ★ | 7 |
| Zheng [15] | ★ | ★ | ★ | ★ | ★ | ★ | ★ | ★ | 8 |
| Kohada [26] | ★ | ★ | ★ | ★ | - | ★ | ★ | ★ | 7 |
| Nishikawa [27] | ★ | ★ | ★ | ★ | - | ★ | ★ | ★ | 7 |
| Son [28] | ★ | ★ | ★ | ★ | - | ★ | ★ | ★ | 7 |
| Tan [29] | ★ | ★ | ★ | ★ | - | ★ | ★ | ★ | 7 |
| Zhang [30] | ★ | ★ | ★ | ★ | - | ★ | ★ | ★ | 7 |
| Altan [16] | ★ | ★ | ★ | ★ | - | - | ★ | ★ | 6 |
| Dalpiaz [17] | ★ | ★ | ★ | ★ | - | ★ | ★ | ★ | 7 |
| Huang [31] | ★ | ★ | ★ | ★ | - | ★ | ★ | ★ | 7 |
| Jiang [32] | - | ★ | ★ | ★ | - | ★ | ★ | ★ | 6 |
| Kang [18] | ★ | ★ | ★ | ★ | - | - | ★ | ★ | 6 |
| Vartolomei [19] | ★ | ★ | ★ | ★ | ★ | ★ | ★ | ★ | 8 |
| Cheng [20] | ★ | ★ | ★ | ★ | - | ★ | ★ | ★ | 7 |
| Huang [33] | ★ | ★ | ★ | ★ | - | ★ | ★ | ★ | 7 |
| Song [34] | ★ | ★ | ★ | ★ | - | ★ | ★ | ★ | 7 |
| Hutterer [35] | ★ | ★ | ★ | ★ | - | ★ | ★ | ★ | 7 |
| Tanaka [36] | ★ | ★ | ★ | ★ | - | ★ | ★ | ★ | 7 |
| Dalpiaz [37] | ★ | ★ | ★ | ★ | ★ | ★ | ★ | ★ | 8 |
| Luo [38] | ★ | ★ | ★ | ★ | - | ★ | ★ | ★ | 7 |
| Tanaka [39] | ★ | ★ | ★ | ★ | - | ★ | ★ | ★ | 7 |
| Azuma [40] | ★ | ★ | ★ | ★ | - | ★ | ★ | ★ | 7 |
| -: The data were not available in this study | | | | | | | | | |
