# Supplementary material for: Prognostic value of preoperative lymphocyte-related systemic inflammatory biomarkers in upper tract urothelial carcinoma patients treated with radical nephroureterectomy: a systematic review and meta-analysis
Source: World J Surg Oncol. 2020 Oct 23;18:273. doi: 10.1186/s12957-020-02048-7 (PMC7585317; doi:10.1186/s12957-020-02048-7)
Supplement: Supplementary file 3 — Additional file 3:. Sensitivity analyses for preoperative NLR in UTUC patients treated with RNU. [file 12957_2020_2048_MOESM3_ESM.docx]

| **Sensitivity analysis for NLR in UTUC patients.** | | | | | |
| --- | --- | --- | --- | --- | --- |
| Study omitted | HR (95% CI) | *P* value | Heterogeneity | | Effect model |
|  |  |  | I^2^ (%) | Ph |  |
| **NLR and OS** |  |  |  |  |  |
| Xu 2020 [24] | 1.58 (1.35-1.85) | <0.001 | 39 | 0.120 | Fixed |
| Jan 2019 [13] | 1.66 (1.44-1.91) | <0.001 | 0 | 0.510 | Fixed |
| Zheng 2019 [15] | 1.65 (1.43-1.90) | <0.001 | 15 | 0.320 | Fixed |
| Tan 2018 [29] | 1.88 (1.30-1.78) | <0.001 | 30 | 0.190 | Fixed |
| Huang 2017 [31] | 1.61 (1.39-1.87) | <0.001 | 40 | 0.110 | Fixed |
| Kang 2017 [18] | 1.60 (1.40-1.85) | <0.001 | 40 | 0.110 | Fixed |
| Cheng 2016 [20] | 1.60 (1.39-1.85) | <0.001 | 40 | 0.110 | Fixed |
| Huang 2016 [33] | 1.61 (1.38-1.87) | <0.001 | 40 | 0.110 | Fixed |
| Dalpiaz 2014 [37] | 1.57 (1.36-1.81) | <0.001 | 28 | 0.200 | Fixed |
| Combined | 1.60 (1.40-1.84) | <0.001 | 31 | 0.170 | Fixed |
| **NLR and CSS** |  |  |  |  |  |
| Xu 2020 [24] | 1.65 (1.36-2.00) | <0.001 | 54 | 0.005 | Random |
| Jan 2019 [13] | 1.69 (1.40-2.03) | <0.001 | 55 | 0.004 | Random |
| Kuroda 2019 [14] | 1.66 (1.38-2.00) | <0.001 | 56 | 0.003 | Random |
| Zheng 2019 [15] | 1.71 (1.43-2.03) | <0.001 | 50 | 0.010 | Random |
| Kohoda 2018 [26] | 1.62 (1.36-1.93) | <0.001 | 52 | 0.009 | Random |
| Son 2018 [28] | 1.66 (1.37-2.01) | <0.001 | 56 | 0.004 | Random |
| Tan 2018 [29] | 1.63 (1.35-1.97) | <0.001 | 52 | 0.008 | Random |
| Huang 2017 [31] | 1.66 (1.37-2.01) | <0.001 | 55 | 0.004 | Random |
| Kang 2017 [18] | 1.67 (1.39-2.01) | <0.001 | 56 | 0.003 | Random |
| Vartolomei 2017 [19] | 1.75 (1.54-1.98) | <0.001 | 18 | 0.250 | Fixed |
| Cheng 2016 [20] | 1.67 (1.39-2.02) | <0.001 | 56 | 0.004 | Random |
| Huang 2016 [33] | 1.66 (1.37-2.01) | <0.001 | 55 | 0.004 | Random |
| Tanaka 2015 [36] | 1.66 (1.37-2.01) | <0.001 | 55 | 0.004 | Random |
| Dalpiaz 2014 [37] | 1.62 (1.36-1.69) | <0.001 | 53 | 0.007 | Random |
| Luo 2014 [38] | 1.52 (1.36-1.69) | <0.001 | 49 | 0.010 | Fixed |
| Tanaka 2014 [39] | 1.68 (1.38-2.05) | <0.001 | 56 | 0.004 | Random |
| Azuma 2013 [40] | 1.61 (1.35-1.92) | <0.001 | 51 | 0.010 | Random |
| Combined | 1.66 (1.39-1.98) | <0.001 | 53 | 0.006 | Random |
| **NLR and DFS/RFS/MFS** |  |  |  |  |  |
| Xu 2020 [24] | 1.60 (1.36-1.89) | <0.001 | 51 | 0.020 | Random |
| Kuroda 2019 [14] | 1.59 (1.37-1.86) | <0.001 | 52 | 0.010 | Random |
| Zheng 2019 [15] | 1.44 (1.31-1.58) | <0.001 | 49 | 0.020 | Fixed |
| Kohada 2019 [26] | 1.44 (1.31-1.58) | <0.001 | 49 | 0.020 | Fixed |
| Nishikawa 2018 [27] | 1.42 (1.30-1.56) | <0.001 | 45 | 0.040 | Fixed |
| Son 2018 [28] | 1.64 (1.38-1.95) | <0.001 | 52 | 0.020 | Random |
| Tan 2018 [29] | 1.41 (1.28-1.56) | <0.001 | 49 | 0.020 | Fixed |
| Altan 2017 [16] | 1.57 (1.35-1.84) | <0.001 | 50 | 0.020 | Random |
| Vartolomei 2017 [19] | 1.60 (1.44-1.78) | <0.001 | 0 | 0.590 | Fixed |
| Song 2016 [34] | 1.44 (1.31-1.58) | <0.001 | 48 | 0.030 | Fixed |
| Tanaka 2015 [36] | 1.60 (1.37-1.88) | <0.001 | 52 | 0.010 | Random |
| Luo 2014 [38] | 1.44 (1.31-1.58) | <0.001 | 48 | 0.030 | Fixed |
| Tanaka 2014 [39] | 1.62 (1.38-1.91) | <0.001 | 52 | 0.010 | Random |
| Azuma 2013 [40] | 1.57 (1.35-1.82) | <0.001 | 50 | 0.020 | Random |
| Combined | 1.45 (1.32-1.59) | <0.001 | 48 | 0.020 | Fixed |
| **NLR and PFS** |  |  |  |  |  |
| Jan 2019 [13] | 3.24 (1.79-5.85) | <0.001 | 0 | 0.660 | Fixed |
| Altan 2017 [16] | 2.11 (0.81-5.47) | 0.13 | 73 | 0.050 | Random |
| Song 2016 [34] | 1.87 (0.94-3.72) | 0.08 | 64 | 0.090 | Random |
| Combined | 2.25 (1.18-4.32) | 0.01 | 65 | 0.060 | Random |
