# Supplementary figures and images for: Prognostic value of preoperative lymphocyte-related systemic inflammatory biomarkers in upper tract urothelial carcinoma patients treated with radical nephroureterectomy: a systematic review and meta-analysis
Source: World J Surg Oncol. 2020 Oct 23;18:273. doi: 10.1186/s12957-020-02048-7 (PMC7585317; doi:10.1186/s12957-020-02048-7)

A    NLR and OS

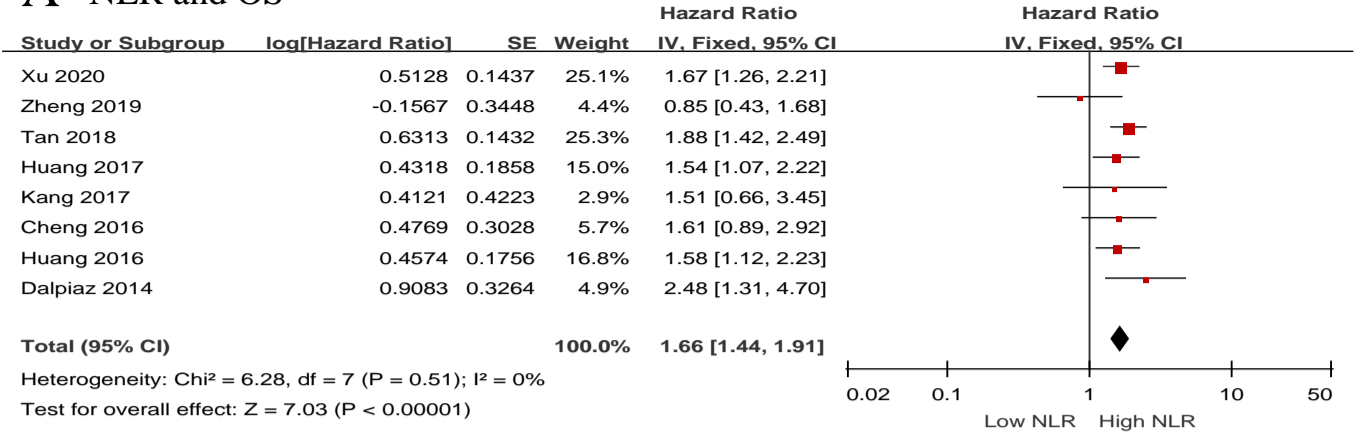

B    NLR and CSS

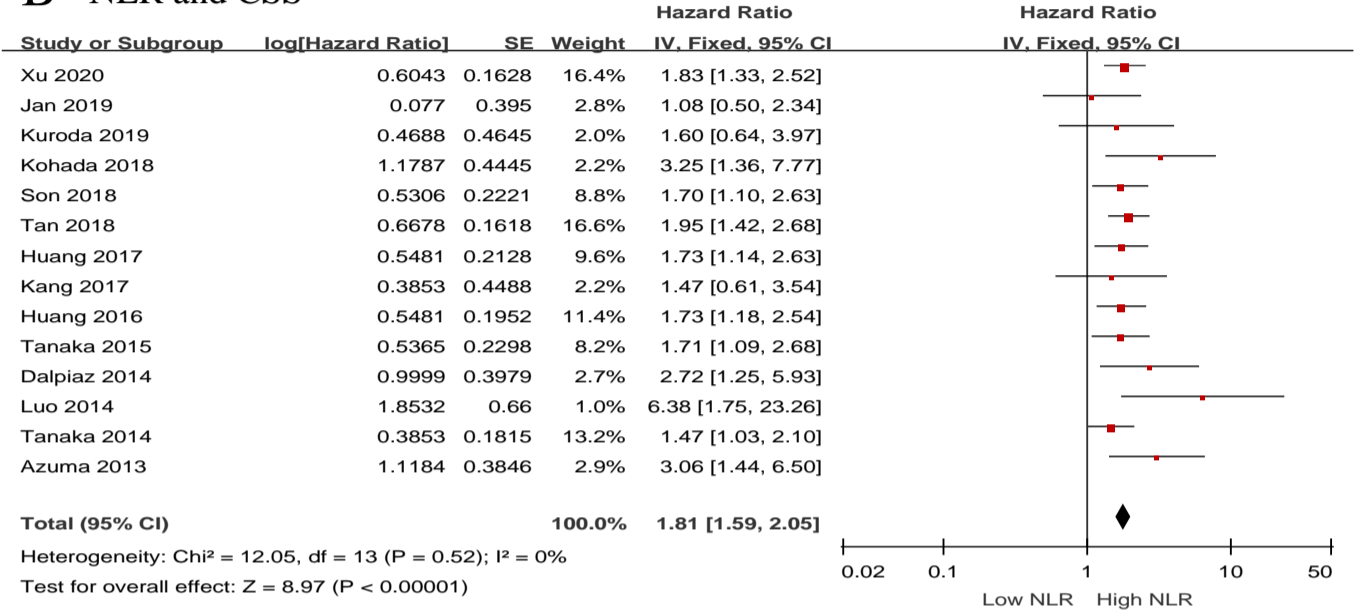

C    NLR and DFS/RFS/MFS

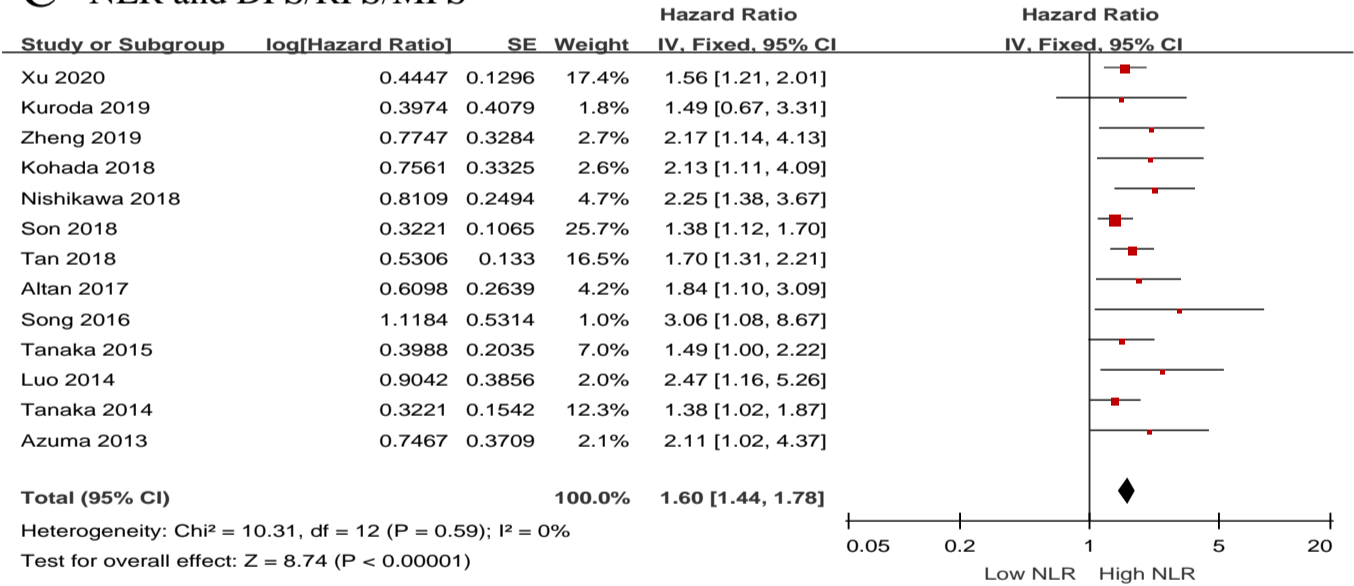

D    NLR and PFS

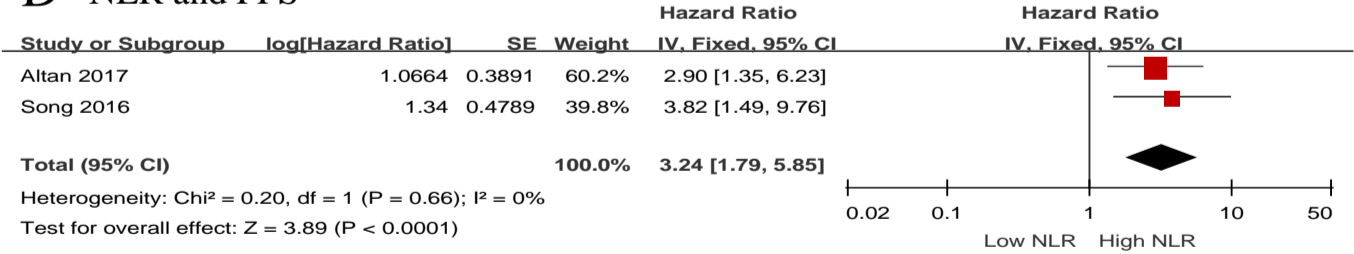

Supplement: Supplementary file 4 — Additional file 4:. Forest plots of the association between NLR and OS, CSS, DFS/RFS/MFS, and PFS after removing the three studies over the pseudo 95% CI according to funnel plot. [file 12957_2020_2048_MOESM4_ESM.pdf]

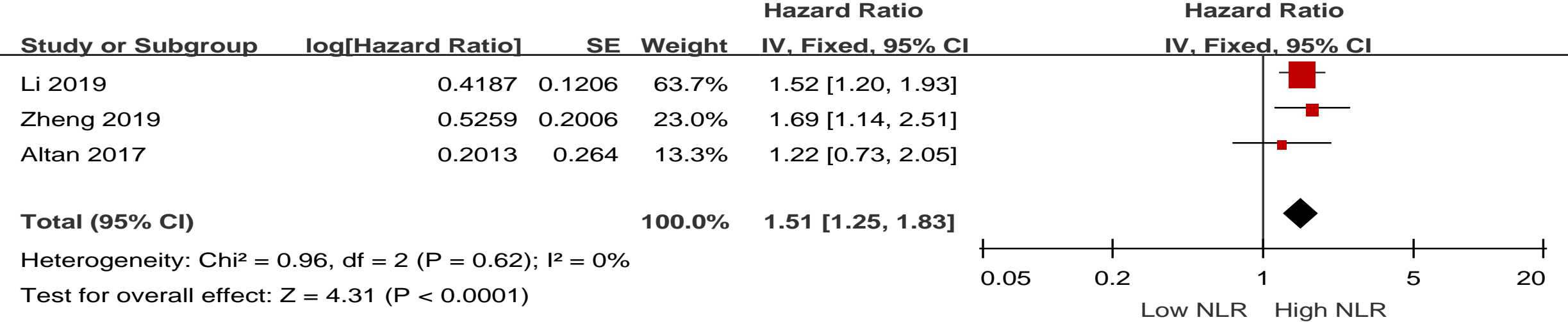

Supplement: Supplementary file 7 — Additional file 7:. Forest plots of the association between MLR and DFS/RFS/MFS after removing the three studies over the pseudo 95% CI according to funnel plot. [file 12957_2020_2048_MOESM7_ESM.pdf]
