# Supplementary material for: Prognostic value of preoperative lymphocyte-related systemic inflammatory biomarkers in upper tract urothelial carcinoma patients treated with radical nephroureterectomy: a systematic review and meta-analysis
Source: World J Surg Oncol. 2020 Oct 23;18:273. doi: 10.1186/s12957-020-02048-7 (PMC7585317; doi:10.1186/s12957-020-02048-7)
Supplement: Supplementary file 5 — Additional file 5:. Sensitivity analyses for preoperative PLR in UTUC patients treated with RNU. [file 12957_2020_2048_MOESM5_ESM.docx]

| **Sensitivity analysis for PLR in UTUC patients.** | | | | | |
| --- | --- | --- | --- | --- | --- |
| Study omitted | HR (95% CI) | *P* value | Heterogeneity | | Effect model |
|  |  |  | I^2^ (%) | Ph |  |
| **PLR and OS** |  |  |  |  |  |
| Jan 2019 [13] | 1.72 (1.26-2.36) | <0.001 | 0 | 0.96 | Fixed |
| Zheng 2019 [15] | 1.46 (1.06-2.02) | =0.020 | 4 | 0.35 | Fixed |
| Dalpiaz 2017 [17] | 1.46 (1.05-2.02) | =0.020 | 2 | 0.36 | Fixed |
| Huang 2016 [33] | 1.51 (1.09-2.10) | =0.010 | 17 | 0.30 | Fixed |
| Combined | 1.54 (1.16-2.04) | =0.003 | 0 | 0.49 | Fixed |
| **PLR and CSS** |  |  |  |  |  |
| Jan 2019 [13] | 1.65 (1.28-2.13) | <0.001 | 0 | 0.72 | Fixed |
| Kuroda 2019 [14] | 1.64 (1.27-2.11) | <0.001 | 0 | 0.61 | Fixed |
| Zheng 2019 [15] | 1.53 (1.18-1.98) | <0.001 | 6 | 0.38 | Fixed |
| Son 2018 [28] | 1.42 (1.07-1.90) | =0.020 | 0 | 0.50 | Fixed |
| Dalpiaz 2017 [17] | 1.49 (1.15-1.92) | =0.003 | 0 | 0.46 | Fixed |
| Jiang 2017 [32] | 1.53 (1.20-1.96) | <0.001 | 4 | 0.39 | Fixed |
| Huang 2016 [33] | 1.55 (1.19-2.01) | <0.001 | 7 | 0.37 | Fixed |
| Combined | 1.55 (1.22-1.96) | <0.001 | 0 | 0.50 | Fixed |
| **PLR and DFS/RFS/MFS** |  |  |  |  |  |
| Kuroda 2019 [14] | 1.36 (1.13-1.63) | <0.001 | 0 | 0.68 | Fixed |
| Zheng 2019 [15] | 1.32 (1.10-1.59) | =0.003 | 0 | 0.40 | Fixed |
| Son 2018 [28] | 1.33 (1.11-1.59) | =0.002 | 0 | 0.40 | Fixed |
| Jiang 2017 [32] | 1.32 (1.11-1.58) | =0.002 | 0 | 0.41 | Fixed |
| Song 2016 [34] | 1.29 (1.08-1.54) | =0.005 | 0 | 0.74 | Fixed |
| Combined | 1.32 (1.11-1.57) | =0.002 | 0 | 0.57 | Fixed |
| **PLR and PFS** |  |  |  |  |  |
| Jan 2019 [13] | 2.21 (1.35-3.61) | =0.002 | 0 | 0.98 | Fixed |
| Altan 2017 [16] | 1.80 (1.29-2.51) | <0.001 | 0 | 0.57 | Fixed |
| Song 2016 [34] | 1.84 (1.34-2.51) | <0.001 | 0 | 0.50 | Fixed |
| Combined | 1.88 (1.41-2.52) | <0.001 | 0 | 0.73 | Fixed |
