# Supplementary material for: Prognostic value of preoperative lymphocyte-related systemic inflammatory biomarkers in upper tract urothelial carcinoma patients treated with radical nephroureterectomy: a systematic review and meta-analysis
Source: World J Surg Oncol. 2020 Oct 23;18:273. doi: 10.1186/s12957-020-02048-7 (PMC7585317; doi:10.1186/s12957-020-02048-7)
Supplement: Supplementary file 6 — Additional file 6:. Sensitivity analyses for preoperative MLR in UTUC patients treated with RNU. [file 12957_2020_2048_MOESM6_ESM.docx]

| **Sensitivity analysis for MLR in UTUC patients.** | | | | | |
| --- | --- | --- | --- | --- | --- |
| Study omitted | HR (95% CI) | *P* value | Heterogeneity | | Effect model |
|  |  |  | I^2^ (%) | Ph |  |
| **MLR and OS** |  |  |  |  |  |
| Jan 2019 [13] | 1.80 (1.49-2.17) | <0.001 | 0 | 0.40 | Fixed |
| Li 2019 [25] | 2.12 (1.64-2.74) | <0.001 | 0 | 0.80 | Fixed |
| Zheng 2019 [15] | 1.76 (1.44-2.15) | <0.001 | 0 | 0.46 | Fixed |
| Zhang 2018 [30] | 1.78 (1.48-2.14) | <0.001 | 0 | 0.56 | Fixed |
| Hutterer 2015 [35] | 1.84 (1.52-2.23) | <0.001 | 11 | 0.34 | Fixed |
| Combined | 1.83 (1.53-2.19) | <0.001 | 0 | 0.50 | Fixed |
| **MLR and CSS** |  |  |  |  |  |
| Jan 2019 [13] | 1.86 (1.47-2.36) | <0.001 | 0 | 0.49 | Fixed |
| Li 2019 [25] | 2.05 (1.39-3.01) | <0.001 | 0 | 0.73 | Fixed |
| Zheng 2019 [15] | 1.78 (1.37-2.31) | <0.001 | 0 | 0.91 | Fixed |
| Combined | 1.86 (1.48-2.33) | <0.001 | 0 | 0.79 | Fixed |
| **MLR and DFS/RFS/MFS** |  |  |  |  |  |
| Li 2019 [25] | 1.90 (1.02-3.53) | =0.04 | 68 | 0.04 | Random |
| Zheng 2019 [15] | 1.75 (1.01-3.05) | =0.05 | 68 | 0.04 | Random |
| Altan 2017 [16] | 1.85 (1.20-2.86) | =0.005 | 63 | 0.07 | Random |
| Song 2016 [34] | 1.51 (1.25-1.83) | <0.001 | 0 | 0.62 | Fixed |
| Combined | 1.65 (1.18-2.30) | =0.003 | 53 | 0.09 | Random |
| **MLR and PFS** |  |  |  |  |  |
| Jan 2019 [13] | 2.88 (1.60-5.19) | <0.001 | 40 | 0.20 | Fixed |
| Altan 2017 [16] | 1.90 (1.02-3.53) | =0.04 | 80 | 0.03 | Random |
| Song 2016 [34] | 1.60 (1.06-2.42) | =0.02 | 0 | 0.32 | Fixed |
| Combined | 2.20 (1.13-4.26) | =0.02 | 61 | 0.08 | Random |
