# Supplementary material for: Prognostic value of preoperative lymphocyte-related systemic inflammatory biomarkers in upper tract urothelial carcinoma patients treated with radical nephroureterectomy: a systematic review and meta-analysis
Source: World J Surg Oncol. 2020 Oct 23;18:273. doi: 10.1186/s12957-020-02048-7 (PMC7585317; doi:10.1186/s12957-020-02048-7)
Supplement: Supplementary file 8 — Additional file 8:. Evaluation of the quality of evidence according to GRADE system. [file 12957_2020_2048_MOESM8_ESM.docx]

| **Additional file 5.** Evaluation of the quality of evidence according to GRADE system | | | | | | | | | | |
| --- | --- | --- | --- | --- | --- | --- | --- | --- | --- | --- |
| Quality assessment | | | | | | | No. of patients | Hazard Ratios (95% CI) | Quality | Importance |
| No. of studies | Design | Risk of bias | Inconsistency | Indirectness | Imprecision | Other considerations |  |  |  |  |
| NLR |  |  |  |  |  |  |  |  |  |  |
| OS |  |  |  |  |  |  |  |  |  |  |
| 9 | observational studies | no serious risk of bias | no serious  inconsistency | no serious  indirectness | no serious imprecision | none | 3496 | 1.60 (1.40-1.84) | Low | Critical |
| CSS |  |  |  |  |  |  |  |  |  |  |
| 17 | observational studies | no serious risk of bias | serious^1^ | no serious  indirectness | no serious imprecision | none | 8672 | 1.66 (1.39-1.98) | Very low | Critical |
| DFS/RFS/MFS |  |  |  |  |  |  |  |  |  |  |
| 14 | observational studies | no serious risk of bias | no serious  inconsistency | no serious  indirectness | no serious imprecision | none | 7243 | 1.45 (1.32-1.59) | Low | Critical |
| PFS |  |  |  |  |  |  |  |  |  |  |
| 3 | observational studies | no serious risk of bias | serious^1^ | no serious  indirectness | no serious imprecision | none | 677 | 2.25 (1.18-4.32) | Very low | Critical |
| PLR |  |  |  |  |  |  |  |  |  |  |
| OS |  |  |  |  |  |  |  |  |  |  |
| 4 | observational studies | no serious risk of bias | no serious  inconsistency | no serious  indirectness | no serious imprecision | none | 1344 | 1.54 (1.16-2.04) | Low | Critical |
| CSS |  |  |  |  |  |  |  |  |  |  |
| 7 | observational studies | no serious risk of bias | no serious  inconsistency | no serious  indirectness | no serious imprecision | none | 2781 | 1.55 (1.22-1.96) | Low | Critical |
| DFS/RFS/MFS |  |  |  |  |  |  |  |  |  |  |
| 5 | observational studies | no serious risk of bias | no serious  inconsistency | no serious  indirectness | no serious imprecision | none | 1836 | 1.32 (1.11-1.57) | Low | Critical |
| PFS |  |  |  |  |  |  |  |  |  |  |
| 3 | observational studies | no serious risk of bias | no serious  inconsistency | no serious  indirectness | no serious imprecision | none | 677 | 1.88 (1.41-2.52) | Low | Critical |
| MLR |  |  |  |  |  |  |  |  |  |  |
| OS |  |  |  |  |  |  |  |  |  |  |
| 5 | observational studies | no serious risk of bias | no serious  inconsistency | no serious  indirectness | no serious imprecision | none | 1669 | 1.83 (1.53-2.19) | Low | Critical |
| CSS |  |  |  |  |  |  |  |  |  |  |
| 3 | observational studies | no serious risk of bias | no serious  inconsistency | no serious  indirectness | no serious imprecision | none | 1387 | 1.86 (1.48-2.33) | Low | Critical |
| DFS/RFS/MFS |  |  |  |  |  |  |  |  |  |  |
| 4 | observational studies | no serious risk of bias | serious^1^ | no serious  indirectness | no serious imprecision | none | 1216 | 1.65 (1.18-2.30) | Very low | Critical |
| PFS |  |  |  |  |  |  |  |  |  |  |
| 3 | observational studies | no serious risk of bias | serious^1^ | no serious  indirectness | no serious imprecision | none | 677 | 2.20 (1.13-4.26) | Very low | Critical |
| ^1^The heterogeneity of this outcome was obvious between studies. | | | | | | | | | | |
